# Intestinal Chelators, Sorbants, and Gut-Derived Uremic Toxins

**DOI:** 10.3390/toxins13020091

**Published:** 2021-01-26

**Authors:** Solène M. Laville, Ziad A. Massy, Said Kamel, Jean Marc Chillon, Gabriel Choukroun, Sophie Liabeuf

**Affiliations:** 1 Centre for Research in Epidemiology and Population Health (CESP), INSERM UMRS 1018, Université Paris-Saclay, F-94807 Villejuif, France; solene.laville@inserm.fr (S.M.L.); ziad.massy@aphp.fr (Z.A.M.); 2 Department of Nephrology, Ambroise Paré University Hospital, APHP, Boulogne Billancourt, F-92100 Paris, France; 3 MP3CV Laboratory, EA7517, Jules Verne University of Picardie, F-80000 Amiens, France; said.kamel@u-picardie.fr (S.K.); jean-marc.chillon@u-picardie.fr (J.M.C.); choukroun.gabriel@chu-amiens.fr (G.C.); 4 Department of Biochemistry, Amiens University Medical Center, F-80000 Amiens, France; 5 Direction of Clinical Research, Amiens University Medical Center, F-80000 Amiens, France; 6 Department of Nephrology Dialysis Transplantation, Amiens University Medical Center, F-80000 Amiens, France; 7 Department of Clinical Pharmacology, Amiens University Medical Center, F-80000 Amiens, France

**Keywords:** uremic toxins, phosphate binders, chronic kidney disease

## Abstract

Chronic kidney disease (CKD) is a highly prevalent condition and is associated with a high comorbidity burden, polymedication, and a high mortality rate. A number of conventional and nonconventional risk factors for comorbidities and mortality in CKD have been identified. Among the nonconventional risk factors, uremic toxins are valuable therapeutic targets. The fact that some uremic toxins are gut-derived suggests that intestinal chelators might have a therapeutic effect. The phosphate binders used to prevent hyperphosphatemia in hemodialysis patients act by complexing inorganic phosphate in the gastrointestinal tract but might conceivably have a nonspecific action on gut-derived uremic toxins. Since phosphorous is a major nutrient for the survival and reproduction of bacteria, changes in its intestinal concentration may impact the gut microbiota’s activity and composition. Furthermore, AST-120 is an orally administered activated charcoal adsorbent that is widely used in Asian countries to specifically decrease uremic toxin levels. In this narrative review, we examine the latest data on the use of oral nonspecific and specific intestinal chelators to reduce levels of gut-derived uremic toxins.

## 1. Introduction

Chronic kidney disease (CKD) is a highly prevalent condition that generates a substantial disease burden worldwide. Over the last few decades, the burden of CKD has not declined to the same extent as it has for many other important noncommunicable diseases [1]. In 2017, an estimated 700 million individuals had CKD (regardless of the stage), more than those affected by diabetes, asthma, chronic obstructive pulmonary disease, or depressive disorders [1]. It was recently shown that patients seen by nephrologists are more difficult to treat than patients seen by other subspecialists, due to the large number of comorbidities, polymedication, and the high mortality risk [2]. 

A number of conventional and nonconventional risk factors for comorbidities and mortality in CKD have been identified. The nonconventional risk factors include uremic toxins, various harmful compounds that accumulate as renal function declines and that are potential therapeutic targets [3,4]. The high comorbidity burden in CKD means that polymedication is common [5,6]. Specific treatment approaches are implemented for complications that can be readily identified and quantified, such as cardiovascular disease, hypertension, anemia, mineral bone disorder, volume overload, electrolyte disorders, and acid-base disorders. At present, there are few specific or nonspecific pharmacological strategies for decreasing uremic toxin levels. The objective of the present narrative review was to assess the efficacy of intestinal chelators (e.g., phosphate binders and the orally administered activated charcoal sorbent AST-120 (Kremezin®, Kureha Corporation, Tokyo, Japan)) for the adsorption of gut-derived uremic toxins. We will describe gut-derived uremic toxins, phosphate binders, and then the effects of phosphate binders and AST-120 on uremic toxin levels.

## 2. Gut-Derived Uremic Toxins

According to the European Society of Artificial Organs’ European Uremic Toxins Work Group [7], uremic toxins are harmful compounds that accumulate in the body during periods of renal function decline. Uremic toxins can be classified according to their molecular weight, water solubility, and protein-binding status. Alternatively, uremic toxins can be classified by their origin: gut-derived or not gut-derived. The present review focused on gut-derived uremic toxins. 

### 2.1. Trimethylamine-N-Oxide

Trimethylamine-N-oxide (TMAO) is a small, water-soluble, gut-derived uremic toxin produced by the oxidation of trimethylamine (TMA). TMAO is derived primarily from dietary choline and carnitine through the action of the gut microbiota, which metabolizes these constituents to TMA. Red meat, eggs, dairy products, and saltwater fish are rich in choline, lecithin, and L-carnitine, and thus constitute potential sources of TMAO [8]. Many bacterial species are involved in producing TMA, including *Clostridia*, *Proteus*, *Shigella*, and *Aerobacter*. Over the last decade, a growing body of preclinical and clinical evidence has identified TMAO as an important contributor to the pathogenesis of cardiovascular disease [9]. Indeed, elevated TMAO levels are associated with a greater incident risk of major cardiovascular adverse events [10,11,12].

### 2.2. Indoxyl Sulfate

Indoxyl sulfate (IS, a protein-binding uremic toxin) is generated after tryptophan present in food is metabolized to indole by intestinal bacteria (mainly *Escherichia coli*). The indole is absorbed by the intestine and then circulates in the blood to the liver. After hydroxylation and sulfation in the liver, indole becomes IS and re-enters the circulation. When the kidney is operating normally, IS in serum enters renal tubular cells via the organic anion transporters (OATs) 1 and 3, located in the proximal basolateral membrane, and is subsequently drained into the renal tubules via OAT4 in the renal tubular cells’ apical membrane [3]. Various studies have suggested that IS is an agonist of the transcription factor aryl hydrocarbon receptor (AhR) and increases oxidative stress and inhibits nitric oxide production in endothelial cells, inhibits neovascularization, and enhances coagulation [3]. In cohort studies of predialysis and dialysis patients, high levels of IS were associated independently with cardiovascular events, renal function decline, and mortality [13,14].

### 2.3. Indole Acetic Acid

Indole-3 acetic acid (IAA) is a protein-bound uremic toxin generated by the metabolism of tryptophan and that belongs to the indole family of uremic solutes (like IS). Serum IAA levels are elevated in patients with CKD. IAA is an agonist of the transcription factor AhR but has been less extensively studied than IS as a uremic toxin. Nevertheless, IAA has been linked to cardiovascular disease via the induction of inflammation and the production of pro-coagulant tissue factors [15]. As with IS, these effects are mainly linked to activation of the AhR, which appears to have a key role in the biological action of several indoles [3,16]. IAA has been linked to elevated mortality and the incidence of cardiovascular events in CKD patients [15].

### 2.4. P-Cresyl Sulfate

P-cresyl sulfate (pCS) is a protein-bound, gut-derived uremic toxin. P-cresol is produced by the bacterial metabolism of tyrosine and phenylalanine in the intestine. Tyrosine and phenylalanine are essential amino acids for human beings and are found in protein-rich foods like meat, dairy products, eggs, and nuts. Conjugation of p-cresol creates pCS and, to a lesser extent, p-cresylglucuronide. The pCS is normally excreted by the kidneys. Data from animal studies suggest that pCS is harmful in several respects. Firstly, pCS contributes to cardiovascular and renal damage. An elevated pCS plasma concentration alters vascular function and in vitro remodeling [4] and is toxic for renal tubular cells and kidney fibrosis [17]. Furthermore, free pCS was found to be a predictor of survival in both dialyzed and nondialyzed CKD patients [14,18,19].

### 2.5. Nonpharmacological Interventions, Dietary Changes, and Uremic Toxin Concentrations

Although this review focused on pharmacological interventions (intestinal chelators) that modulate the concentrations of uremic toxins and prevent the development of harmful effect in CKD patients, diet may also have a role in the therapeutic strategy. Indeed, plant nutrients and plant-based diets can have beneficial effects in patients with CKD [20]. In CKD, there is a direct relationship between the protein/dietary fiber ratio and the IS and pCS levels. Di Iorio et al. demonstrated that nutritional therapy such as Mediterranean diet and very low protein diet (which is a vegetarian diet) are effective in lowering IS and pCS serum levels and ameliorated the intestinal permeability in CKD patients [20]. In patients on maintenance hemodiafiltration, plasma levels of IS and pCS were lower in vegetarian individuals than in nonvegetarian individuals [21]. This finding was confirmed in a randomized controlled trial of synbiotic therapy in nondialysis patients [22]. Likewise, a study of people with normal renal function found that pCS and IS production rates were markedly lower in vegetarians than in individuals consuming an unrestricted diet [23]. Furthermore, vegetarian diets contain less lecithin, choline, and l-carnitine; this might result in less TMAO production [24].

## 3. Phosphate Binders

Several lines of evidence link high phosphate concentrations to adverse health effects in patients with CKD [25,26,27]. Intestinal phosphate binders have been authorized for lowering phosphate concentrations in dialysis patients and for sevelamer carbonate in nondialyzed patients [28]. The phosphate binders act by decreasing the absorption of ingested phosphate and converting it into an insoluble form that is excreted in the stools. This mechanism of action explains why the binders must be taken at the same time as phosphate-containing foods.

There are two categories of phosphate binder: (1) calcium-based binders (calcium carbonate, calcium acetate, and calcium acetate/magnesium carbonate) and (2) noncalcium-based binders (sevelamer, lanthanum, and, more recently, iron-based binders). Although the two categories are equally effective in lowering serum phosphate concentrations when well titrated [29], each has various drawbacks, e.g., a positive calcium balance and potentially harmful cardiovascular outcomes for calcium-based binders [30] and the higher cost of noncalcium-based binders. Furthermore, the pill burden and gastrointestinal side effects associated with phosphate binders are major causes of poor treatment compliance [31,32] (Table 1). 

### 3.1. Calcium-Based Phosphate Binders

For many years, calcium-based phosphate binders constituted the cornerstone of hyperphosphatemia treatment. Although these binders are cheap and effective in lowering serum phosphate in dialysis patients and have a satisfactory safety profile, they have been linked to hypercalcemia (more frequently with calcium acetate than with calcium carbonate [53]) and the progression of vascular calcification [54]. Patients with high calcemia values should not, therefore, be treated with calcium-based phosphate binders [55].

### 3.2. Magnesium-Based Phosphate Binders.

A magnesium–calcium combination is a well-tolerated alternative to calcium acetate alone; it reduces the calcium load in hemodialysis patients and is not inferior to sevelamer hydrochloride [56]. Furthermore, magnesium may be of additional value in CKD patients because it appears to inhibit calcification [57,58,59].

### 3.3. Calcium-Free/Noncalcium-Based Phosphate Binders

Sevelamer was the first metal-free, calcium-free phosphate binder (first as the hydrochloride and then sevelamer carbonate) to become available for the treatment of hyperphosphatemia in patients on dialysis. Several randomized trials have compared sevelamer with calcium-based phosphate binders, particularly with regard to the progression of cardiovascular disease at different CKD stages. The majority of these trials showed that treatment with sevelamer was associated with less vascular calcification and that calcium-based binders were associated with more vascular calcification [34,44,60,61]. Sevelamer appears to induce constipation more frequently than calcium-based lanthanum and iron-based phosphate binders do [29]. 

Lanthanum carbonate is a powerful metal-based but calcium-free phosphate chelator [62]. Concerns have been raised about its safety, especially with regard to possible accumulation in the liver (like aluminum). However, this has not been confirmed in studies in humans [63]. Like most noncalcium binders, lanthanum carbonate is associated with less vascular calcification when compared with calcium-based binders [36]. Although the pill burden is lower than for sevelamer, gastrointestinal effects (such as nausea, vomiting, and abdominal cramps) are common [29,64].

### 3.4. Iron-Based Phosphate Binders

Two iron-based phosphate binders are currently on the market: ferric citrate and sucroferric oxyhydroxide. Both are effective phosphate binders and are not inferior to other compounds in this respect [65,66]. 

Ferric citrate binds phosphate in exchange for citrate to form ferric phosphate, which is insoluble and is excreted in the feces. In one trial, ferric citrate was non-inferior to the comparator arm (sevelamer or calcium-containing) in controlling serum phosphate levels [39]. Furthermore, ferric citrate was associated with an elevation in serum ferritin, which reduced the need for intravenous iron and erythropoietin-stimulating agents and increased hemoglobin levels [39,67,68]. 

Sucroferric oxyhydroxide is a novel, polynuclear, chewable, iron-based phosphate binder that can produce an insoluble complex in the gastrointestinal tract. In a Phase III randomized trial, sucroferric oxyhydroxide was as effective as sevelamer in lowering serum phosphate in dialysis patients, and the pill burden was 75% lower [42]. Furthermore, use of sucroferric oxyhydroxide led to an elevation in serum iron levels, albeit to a much lesser extent than for ferric citrate [43].

Both of these iron-based binders are associated with mild to moderate diarrhea and stool discoloration [29,64].

The 2017 Kidney Disease: Improving Global Outcomes (KDIGO) guidelines recommend “lowering elevated phosphate levels toward the normal range” but do not specify which types of phosphate binder should be used. However, the KDIGO guidelines also suggest avoiding hypercalcemia [55].

Observational studies have shown that the risk of all-cause mortality and cardiovascular disease among dialysis patients is lower among those treated with phosphate binders [69]. A meta-analysis showed that noncalcium chelators (sevelamer and lanthanum) were associated with a lower risk of mortality than calcium chelators (carbonate and calcium acetate) [70]. However, placebo-controlled, randomized trials have not found a beneficial impact of phosphate binders on mortality [29]. However, these trials included small numbers of patients and did not have long-term follow-up. Sevelamer could lead to a reduction in vascular calcification vs. calcium-based phosphate binders [71]. The advantages and disadvantages of phosphate chelators are summarized in Table 1. 

## 4. Phosphate Binders and Gut-Derived Uremic Toxins

### 4.1. Phosphate Binders and the Gut Microflora 

Due to the diversity and adaptability of the gut microbiome, changes in diet can quickly and dramatically influence the species of bacteria present. Thus, altering the concentrations and effects of bacterial metabolites might influence the host’s metabolism [72,73]. CKD patients (especially those on dialysis) have to follow a low-phosphorus, low-potassium diet due to the association between high serum levels of these metabolites and the mortality risk [74]. Since phosphorous is a major nutrient for the survival and reproduction of bacteria, changes in its intestinal concentration may impact the gut microbiota’s activity and composition [73]. Therefore, these dietary restrictions alter the composition of the gut microbiome. Hence, the gut microbiome is influenced not only by CKD but also by phosphate binders [75,76].

### 4.2. Phosphate Binders and Gut-Derived Uremic Toxins

#### 4.2.1. Experimental Data

The phosphate binders’ impact on the gut microflora can potentially modify the generation of gut-derived uremic toxins. Phosphate binders might prevent the absorption of both phosphate and gut-derived uremic toxins. There are few published in vitro studies of the impact of phosphate binders on precursors or uremic toxins present in the intestine. A recent report (published as a poster only) suggested that sevelamer hydrochloride induced a decrease in levels of indole and p-cresol (the gut precursors of pCS and IS) [77]. Another recent report indicated that sevelamer carbonate was able to adsorb IAA but not indole and p-cresol. The results can, perhaps, be explained by the compounds’ respective chemical structures: IAA has a carboxylic acid group (like bile acids, which are also chelated by sevelamer) but none of the precursors have functional groups that would enable chelation by sevelamer [78] (Table 2). 

In a study of uremic apolipoprotein-E-deficient mice, the serum concentrations of IS and IAA did not fall significantly, in contrast to the serum phosphorus concentration, after an eight-week sevelamer-containing diet [79].

Clinical studies of the impact of phosphate binders on gut-derived uremic toxin concentrations are summarized in Table 2.

#### 4.2.2. Observational Studies

In an observational, noncomparative study of five hemodialysis patients, Lin et al. evaluated serum levels of IS and pCS after 12 weeks of sevelamer hydrochloride treatment. A significant decrease was seen for levels of pCS, when a nonsignificant trend was observed for IS levels [81]. In a cross-sectional, noncomparative, observational study, Guida et al. assessed the serum pCS levels in a total of 57 patients on peritoneal dialysis. Of the 45 patients with hyperphosphatemia, 29 received sevelamer hydrochloride and 16 were treated with another phosphate binder (lanthanum). Patients with normal phosphatemia were not treated (*n* = 12). Plasma p-cresol concentrations were significantly lower in patients on sevelamer than in patients receiving lanthanum or those not treated. [80]. To note, the circulating toxin is pCS and not p-cresol; the latter is essentially generated ex vivo during the processing of phenol-containing blood samples [86].

Recently, a study in 18 hemodialysis patients found that 12 weeks of treatment with sucroferric oxyhydroxide was associated with higher IS and pCS concentrations [76]. On the same lines, the results of Dai et al.’s observational study of 423 patients with end-stage renal disease showed that serum levels of IS and TMAO (but not pCS) were significantly higher in sevelamer users than in non-users [82]. However, the observational design of these two recent studies constitutes an important limitation on the interpretation of the results; indication bias might account for the elevated levels of gut-derived uremic toxins observed in the sevelamer groups. The data from the interventional studies described below appear to be more robust.

#### 4.2.3. Interventional Studies

An interventional, controlled, crossover study included 57 hemodialysis patients, during which they received an eight-week treatment of sevelamer hydrochloride. No changes in serum levels were observed for IS and IAA; unexpectedly, the serum concentration of pCS increased significantly [83]. 

In nondialyzed patients, in a single-blind, placebo-controlled, randomized clinical trial, plasma levels of p-cresol decreased significantly in the sevelamer group only [84]. In a recent, multicenter, comparative, randomized, clinical trial in dialysis patients, neither sevelamer nor nicotinamide induced a change in gut-derived uremic toxin levels [85]. Likewise, in a multicenter, double-blind, placebo-controlled, randomized, clinical trial in grades 3 and 4 CKD patients, a three-month course of sevelamer carbonate was not associated with significant changes in the serum concentrations of pCS, IS, or IAA [78].

Hence, in view of the literature data from interventional studies, sevelamer has no real effect on gut-derived uremic toxins. 

## 5. Sorbents and Uremic Toxins

AST-120 is an oral-activated, charcoal adsorbent consisting of water-insoluble porous carbon particles (diameter: 0.2–0.4 mm). The adsorbent has been commercialized since 1991 in Japan, since 2004 in Korea, since 2007 in Taiwan, and since 2010 in the Philippines. It is indicated for the treatment of symptoms of uremia and to prolong the time to dialysis initiation in patients with progressive CKD [87].

In mechanistic terms, AST-120 might act by adsorbing uremic toxins and their precursors in the gastrointestinal tract, allowing them to be excreted in the feces before they can be absorbed into the bloodstream. AST-120 can adsorb the IS and pCS precursors generated by amino acid metabolism in the intestine. Most of the data on potential decreases in gut-derived uremic toxin levels by AST-120 concern IS. Administration of IS increases oxidative stress in the rat kidney, whereas co-administration of AST-120 reduced oxidative stress in CKD rats [88]. In an experimental model of apoE-/- mice with CKD, IS appears to be an important contributor to the vascular dysfunction, and AST-120 treatment ameliorates this dysfunction, possibly via a decrease in serum IS concentration [89]. In a dose-ranging study in 164 CKD patients in the USA, AST-120 decreased serum IS levels in a dose-dependent fashion [90]. In the Kremezin Study against Renal Disease Progression performed in 579 grades 3 and 4 CKD patients in Korea, the AST-120-induced decrease in the serum IS concentration was inversely correlated with the occurrence of the composite primary outcome (progression of renal disease) [91]. 

From 2007 to 2012, the multinational, randomized, double-blind, placebo-controlled Evaluating Prevention of Progression in CKD 1 and 2 trials were conducted in North America, Latin America, and Europe. The objective was to evaluate the effects of add-on AST-120 therapy (vs. placebo) on CKD progression [92]. The primary endpoint was a composite of serum creatinine doubling and number of dialysis initiation and kidney transplantation. The time to the primary endpoint was similar in the AST-120 and placebo groups in each trial individually and in a pooled analysis of the two trials. However, the serum levels of IS (a guide to the patients’ treatment compliance) were not evaluated. Indeed, in a post hoc analysis of the patients in the USA, there was a significant intergroup difference in the time to achieve the primary endpoint (hazard ratio (95% confidence interval) = 0.74 )0.56–0.97) in the per-protocol population, with compliance rates of ≥67%) [93].

The majority of clinical trials on AST-120 have been performed in Asia and essentially focused on kidney disease progression. As this compound seems to effectively reduce gut-derived uremic toxin levels and given the organ toxicity of these toxins, there is a need for new studies of AST-120’s effects on other outcomes (e.g., patient-reported outcomes, such as symptoms potentially due to the accumulation of uremic toxins).

## 6. Conclusions

Even though gut-derived uremic toxins appear to be valuable therapeutic targets, repositioned phosphate binders do not appear to effectively decrease circulating levels of these toxic compounds. AST-120, a specific uremic toxin sorbent only commercialized in Asia, is effective in reducing uremic toxin levels, mostly IS. However, the sorbent’s putative effectiveness on clinical outcomes and mortality must now be assessed in further trials.

## Figures and Tables

**Table 1:** Advantages and disadvantages of various phosphate binders.

	Calcium-Based Phosphate Binders	Magnesium/Calcium-Based Phosphate Binders	Calcium-Free Phosphate Binders			
	*Calcium carbonate* *Calcium acetate*	*Magnesium carbonate*	*Sevelamer*	*Lanthanum carbonate*	*Iron-Based Phosphate Binders*	
					*Ferric citrate*	*Sucroferric oxyhydroxide*
**Advantages**	- Correction of hypocalcemia - Low cost	- Low cost - Potential benefit on vascular calcification [33]	- No systemic absorption - No hypercalcemia [29] - Reduction in vascular calcification vs. calcium-based phosphate binders [34] - Reduction in LDL-cholesterol [35]	- Low pill burden - Reduction in vascular calcification vs. calcium-based phosphate binders [36,37]	- Beneficial effect on iron-deficiency anemia [38,39]	- Low pill burden [40,41,42] - Low systemic absorption [43]
**Disadvantages**	- Moderate pill burden - Systemic absorption - Positive calcium balance (hypercalcemia) - Progression of extraskeletal calcification (vascular calcification [44])	- High pill burden	- High pill burden - High cost - Reduction in intestinal absorption of certain drugs and vitamins [45] - Gastrointestinal adverse events (constipation) - Gastropathy (sevelamer crystals and mucosa injury) [46,47,48]	- High cost - Difficult to chew - Gastrointestinal adverse events - Lanthanum gastropathy (gastrointestinal deposits) [49,50,51,52]	- High pill burden - High cost - Potential iron overload - Gastrointestinal adverse events (diarrhea)	- High cost - Gastrointestinal adverse events

**Table 2:** Summary of preclinical and clinical studies of the impact of phosphate binders on concentrations of gut-derived uremic toxins

**In Vitro Studies**					
**First Author** **Year**	**Type of Binder**		**Type of Gut-Derived Uremic Toxin**	**Results**	
De Smet R. 2016 (abstract) [77]	Sevelamer hydrochloride		IAA, p-cresol, indole	Sevelamer hydrochloride was able to adsorb indole, IAA and p-cresol	
Bennis Y. 2019 [78]	Sevelamer carbonate		IAA, p-cresol, indole	Sevelamer carbonate was able to adsorb IAA but not indole or p-cresol	
**Animal Studies**					
**First Author** **Year**	**Type of Models**		**Type of Binder**	**Type of Gut-Derived Uremic Toxin**	**Results**
Phan O. 2005 [79]	apolipoprotein E–deficient mice		8 weeks of a sevelamer-containing diet	ISI AA	Levels of IS and IAA had not decreased significantly after 8 weeks of a sevelamer-containing diet
**Clinical Studies**					
**First Author** **Year**	**Type of Study**	**Patient Type and Numbers**	**Type of Phosphate Binder**	**Type of Gut-Derived Uremic Toxin**	**Results**
**Observational Studies**					
Guida B. 2013 [80]	Cross-sectional observational study	57 patients on peritoneal dialysis	Sevelamer (*n* = 29) Lanthanum (*n* = 16) No binders (*n* = 12)	P-cresol	Patients on sevelamer had p-cresol levels significantly lower than those receiving lanthanum or no drug
Lin C. 2017 [81]	Observational Noncomparative	5 hemodialysis patients	Sevelamer for 3 months	IS pCS	Significant Reduction in pCS but not IS
Iguchi A. 2020 [76]	Observational cohort	18 hemodialysis patients	Sucroferric oxyhydroxide for 3 months	IS pCS	Increase levels of IS and pCS
Dai L. 2020 [82]	Cross-sectional observational cohort	423 ESKD patients	Calcium-containing phosphate binders Sevelamer	IS pCS TMAO	Increased levels of IS and TMAO, no change in pCS in sevelamer users vs. sevelamer non-users
**Interventional studies**					
Brandenburg V.M. 2010 [83]	Clinical trial (controlled crossover study)	57 hemodialysis patients	3-phase trial (A-B-A design; 8 weeks per phase). Sevelamer was only administered in the middle phase of the study.	ISI AA p-cresol	No impact on IS and IAA levels and a significant rise in p-cresol during the sevelamer period
Riccio E. 2018 [84]	Clinical trial (single-blind, placebo-controlled randomized trial)	69 CKD patients (grade 3–5, not on dialysis)	Sevelamer vs. placebo for 3 months	p-cresol	Significant Reduction in p-cresol after 3 months of treatment by sevelamer but not placebo
Bennis Y. 2019 [78]	Clinical trial (multicenter, double-blind, placebo-controlled, randomized)	78 CKD patients (grade 3–4)	Sevelamer vs. placebo for 3 months	IS pCS IAA	No significant changes in IS, pCS and IAA levels in the sevelamer arm
Lenglet A. 2019 [85]	Clinical trial (multicenter, open-label, randomized controlled trial)	100 hemodialysis patients	Sevelamer vs. nicotinamide	IS pCS TMAO	No impact on IS, pCS or TMAO levels in either arm

ESKD: end-stage kidney disease.
